# Long-term outcomes following severe COVID-19 infection: a propensity matched cohort study

**DOI:** 10.1136/bmjresp-2021-001080

**Published:** 2021-12-09

**Authors:** Joanne McPeake, Martin Shaw, Pamela MacTavish, Kevin G Blyth, Helen Devine, Gillian Fleming, Justine Griffin, Lisa Gemmell, Pauline Grose, Mark Henderson, Philip Henderson, Lucy Hogg, Kirstin King, Iain McInnes, Peter O'Brien, Kathryn Puxty, Callum Rainey, Varun Sharma, Malcolm Sim, Laura Strachan, Stefan Siebert, Tara Quasim

**Affiliations:** 1 NHS Greater Glasgow and Clyde, Glasgow, UK; 2 School of Medicine, Dentistry and Nursing, University of Glasgow, Glasgow, UK; 3 Institute of Cancer Sciences, University of Glasgow, Glasgow, UK; 4 NHS Fife, Kirkcaldy, UK; 5 University of Glasgow, College of Medical, Veterinary and Life Science, Glasgow, UK; 6 NHS Ayrshire and Arran, Ayr, UK; 7 Institute of Infection, Immunity and Inflammation, University of Glasgow, Glasgow, UK

**Keywords:** ARDS, COVID-19

## Abstract

**Background:**

There are limited data describing the long-term outcomes of severe COVID-19. We aimed to evaluate the long-term psychosocial and physical consequences of severe COVID-19 for patients.

**Methods:**

We conducted a multicentre observational cohort study; between 3 and 7 months posthospital discharge, patients who had been admitted to critical care due to severe COVID-19 were invited to an established recovery service. Standardised questionnaires concerning emotional, physical and social recovery, including information on employment, were completed by patients. Using propensity score matching, we explored outcomes between patients admitted to critical care with and without COVID-19, using data from the same recovery programme.

**Results:**

Between July 2020 and December 2020, 93 patients who had been admitted to critical with COVID-19 participated. Emotional dysfunction was common: 46.2% of patients had symptoms of anxiety and 34.4% symptoms of depression. At follow-up 53.7% of previously employed patients had returned to employment; there was a significant difference in return to employment across the socio-economic gradient, with lower numbers of patients from the most deprived areas returning to employment (p=0.03). 91 (97.8%) COVID-19 patients were matched with 91 non-COVID-19 patients. There were no significant differences in any measured outcomes between the two cohorts.

**Interpretation:**

Emotional and social problems are common in survivors of severe COVID-19 infection. Coordinated rehabilitation is required to ensure patients make an optimal recovery.

**Key messages**

- Following severe COVID-19 infection, patients commonly experience emotional problems such as anxiety and depression.
- In this cohort study, patients residing in the most deprived communities were less likely to return to employment, in comparison to those from the least deprived communities.
- Coordinated rehabilitation is required to ensure that both patients and family members make an optimal recovery.

## Introduction

The COVID-19 pandemic has had a profound impact on critical care services.^1 2^ Of those patients who require acute hospitalisation, 14%–18% require admission to a critical care unit.^3 4^ The clinical presentation of COVID-19 and its sequelae make delivery of person-centred care challenging, with limited interaction with family members due to restrictions on hospital visitation during often protracted critical care and hospital stays.^5 6^

Patients who have been critically ill are at high risk of developing physical, psychosocial and cognitive problems following discharge from hospital.^7–11^ These postdischarge issues are known to have a negative influence on societal reintegration for the patient, as well as impacting their close family members.^12 13^ However, there are minimal data describing the long-term outcomes of those who have been severely ill due to COVID-19, and the impact that this will have on wider social outcomes such as return to employment.

The aims of this multicentre cohort study were twofold: (1) Understand the long-term psychosocial and physical consequences, including impact on employment, of severe COVID-19 infection and (2) Explore if critically ill COVID-19 survivors have unique long-term outcomes, in relation to patients admitted to critical care without COVID-19.

## Methods

### Design

We undertook a multicentre, prospective observational cohort study. Results are reported per the Strengthening the Reporting of Observational Studies in Epidemiology (STROBE) guidelines.^14^ All patients provided consent.

We compared the incidence of post hospital problems in patients with a diagnosis of severe COVID-19 pneumonia (COVID-19 cohort) versus critically ill patients without COVID-19 pneumonia (non-COVID-19 cohort) using a propensity score matching analysis.

### Participants

#### COVID-19 cohort

Patients who had been admitted to one of seven critical care units, in five hospitals across Scotland between March 2020 and May 2020 with SARS-CoV-2(or a high clinical suspicion of SARS-CoV-2) were invited to a pre-existing Intensive Care Unit (ICU) rehabilitation programme, 3–7 months posthospital discharge. These sites represent a mix of inner-city tertiary referral hospitals and district general hospitals. A critical care unit in this study delivered either level two or three care, as defined by the UK Intensive Care Society.^15^

### Non-COVID-19 cohort

Patients who had been admitted to one of five critical care units, in five hospitals across Scotland, were invited to the same ICU rehabilitation programme (8–15 weeks posthospital discharge), between May 2016 and October 2018. In total, 206 patients consented to inclusion in this cohort. Due to missing data, we undertook propensity score matching with 182 patients. Information on missing data is shown in online supplemental file S1.

10.1136/bmjresp-2021-001080.supp1 Supplementary data

### Setting

All patients were recruited from an established critical care follow- up programme. Details of the rehabilitation programme, Intensive Care Syndrome: Promoting Independence and Return to Employment (InS:PIRE), have been published previously.^16 17^ Briefly, patients are reviewed by the critical care multidisciplinary team, including nurses, medical staff, pharmacists and physiotherapists, after discharge from critical care. Onward referral to other services such as welfare support, dietetics and clinical psychology are available. The InS:PIRE service also includes specific support about return to employment and vocational rehabilitation.

Due to hospital attendance restrictions in place across the UK, all clinic consultations for the COVID-19 cohort took place virtually or by telephone. Due to the long-term respiratory complications which are predicted in survivors of severe COVID-19, the COVID-19 cohort were also part of an integrated respiratory referral pathway.

### Data collection

All patients were invited to take part in this study during their appointment (in-person for the non-COVID-19 cohort, and virtually for the COVID-19 cohort). If agreeable to research participation, the patient was contacted by a member of the research team (also members of the direct clinical care team) and data for this study was obtained. All data collection was undertaken via telephone or through postal completion of the questionnaires for the COVID-19 cohort, or in-person for the non-COVID-19 cohort. Study outcome measures were obtained before any referrals arising from the consultation had taken place (ie, vocational rehabilitation).

Patient demographic and clinical data was obtained from clinical notes and discharge summaries. Comorbidity data (including mental health data) were obtained from medical notes and critical care admission records. Critical care length of stay was taken for the highest level of care, during the first critical care admission only.

The Scottish Index of Multiple Deprivation (SIMD) is produced by the Scottish Government as a measure of deprivation, with postcode areas defining data on socioeconomic status. This research split the SIMD into five categories to define socioeconomic status; quintile one represented the most deprived and quintile five the least.^18^

## Patient and public involvement

The InS:PIRE service was coproduced with survivors of critical illness and their family members. We designed the intervention and all outcome measures with the previous service users through the creation of a patient and family advisory council. Throughout both the feasibility work and the scale up work of the InS:PIRE programme, priority of the research question, choice of outcome measures, and methods of recruitment were informed by further structured, service user feedback.

## Outcome measures

### Patient outcomes

We sought to understand how baseline employment status had been impacted by severe COVID-19 infection. Employment data were collected during the clinical consultation. We used four potential categories for employment status: employed; not employed; retired and unknown. This preplanned analysis specifically sought to delineate the impact of deprivation on return to employment. Employment data were not collected for the non-COVID-19 cohort at this timepoint, thus, was not included as part of the Propensity Score Match analyses.

Health-related quality of life (HRQoL) was measured using the EQ-5D-5L tool (EuroQuality of Life Group).^19^ The measure comprises two sections: a five-question descriptive component which explores health domains (mobility, self-care, ability to undertake usual activities, pain and mental health) and a Visual Analogue Scale about HRQoL. Each of five questions has five possible answers. These answers equate to a five-digit sequence which is then used to determine a Health Utility Score (HUS). In EQ-5D evaluations, a HUS of 1 equates to the best health state possible, 0 with death and a negative HUS equates to a state worse than death.

Emotional health was measured using the Hospital Anxiety and Depression Score (HADS).^20^ The HADS questionnaire contains 14 statements relating to mood, with 7 statements relating to depression and seven to anxiety. Each statement has four potential options (scored 0–3). Online supplemental file S2 describes the cut-off points utilised for scores obtained from the HADS. Both the HADS and EQ-5D-5L have been recommended as core outcome measures in acute respiratory failure research.^21^ Appropriate licensing requirements were in place for the HADS and EQ-5D-5L.

10.1136/bmjresp-2021-001080.supp2 Supplementary data

Information about ongoing pain was obtained via the Brief Pain Inventory (BPI).^22^ Participants identified where the most severe area of pain was, as well as any other painful sites. Using the BPI pain intensity, alongside interference with function (for example household activities, walking, sleeping and mood) was assessed. Each item was rated on an 11-point ordinal scale (0=no pain and 10=worst pain). Participants were asked to describe new pain (since hospital discharge) only. Information about symptoms of breathlessness and fatigue were obtained with fixed single questions.

## Statistical analysis

Analyses was undertaken using R (V.4.0.5). All missing covariates were imputed for analysis using predictive mean matching with the Multivariate Imputation by Chained Equations software package. Each variable with missing values was regressed on all other analysed variables per single imputation. We did not input return to employment data. Information on missing data for both cohorts included in this study is presented in online supplemental file S1. Employment, breathlessness and fatigue data was not available for the non-COVID-19 cohort.

Continuous variables were expressed as medians and IQR. The Kruskall-Wallis test was used to compare different subgroups and the χ^2^ test to analyse categorical variables.

Logistic regression was used to examine the impact of deprivation on failure to return to employment. SIMD was encoded in a categorical fashion, which allowed the estimation of a non-linear effect between the sociogradient and return to employment. Based on previous literature in this field and univariable associations, we adjusted for pre-existing multimorbidity, follow-up time, age and critical care length of stay.^23^

Propensity score matching was performed matching for: age; gender; socioeconomic deprivation; hospital site; critical care length of stay; Acute Physiology and Chronic Health Evaluation II (APACHE II); time to follow-up; presence of obesity or mental issue pre-ICU and presence of multimorbidity (two or more comorbidities). The pain/discomfort analysis, which was undertaken with the propensity score matched data, was undertaken using logistic regression, dichotomised at severe pain and above in the EQ-5D-5L scale. Four linear regression models were utilised for the remaining outcomes (EQ-5D-5L HUS, EQ-5D-5L Visual Analogue Scale and HADS). Following the propensity score matching analyses we adjusted for the following variables: age; gender; socioeconomic deprivation; ICU length of stay; APACHE II; time to follow-up; presence of obesity or mental issue pre-ICU and presence of multimorbidity (two or more comorbidities). This approach minimised bias during this stage of analysis. To account for any imputational bias during the modelling and matching process, we undertook multiple imputations (10 imputations alongside 10 iterations).

Covariates for each of the statistical approaches (logistic regression and propensity score matching) were based on univariable associations, previous literature in this field and domain expertise. Although the covariate selection is similar across each of the strategies; the propensity score match modelling includes more covariate adjustment, as a larger sample size facilitated this.

## Results

### COVID-19 cohort characteristics

**Figure 1:**
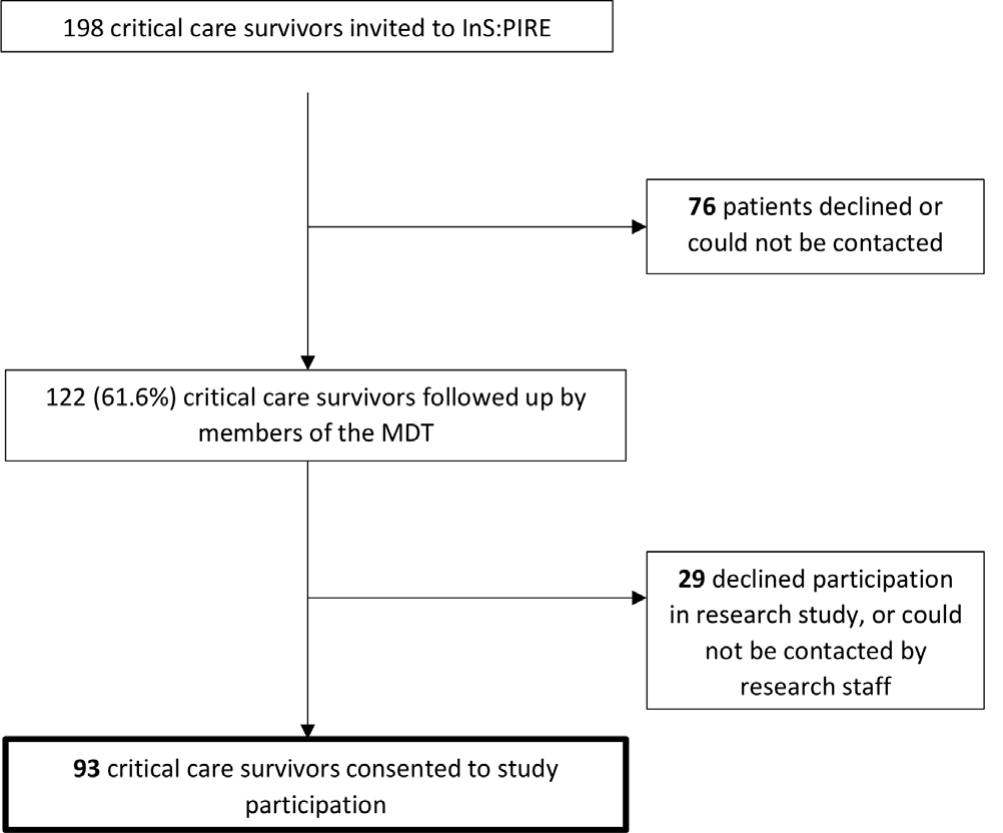
Patient flow through the study (invitation through to participation). InS:PIRE, Intensive Care Syndrome: Promoting Independence and Return to Employment; MDT, multidisciplinary team.

Across the five sites involved, 198 patients who had a diagnosis of COVID-19 were invited to attend follow-up; 122 (61.6%) patients were reviewed and approached about participation in this research; 93 consented to take part (figure 1).

Sixty-one participants (65.6%) were male, the median patient age was 59 (IQR:54–67) years and the median hospital length of stay was 22 (IQR: 12–55.7) days. Sixty-three (67.7%) patients received invasive ventilation and 18 (19.4%) received renal replacement therapy (table 1). The median time to follow-up was 135 (IQR: 85–181) days following hospital discharge. A description of COVID-19 patients who were invited but did not attend is shown in online supplemental file S3.

10.1136/bmjresp-2021-001080.supp3 Supplementary data

**Table 1:** Patient baseline demographics (COVID-19 cohort)

Characteristic	n=93
Gender, male (%)	61 (65.6)
Age, median years (IQR)	59 (54–67)
Obesity (%)	30 (32.3)
Black and minority ethnic (%)	4 (4.3)
Smoking (%)	7 (7.5)
Comorbidities	
Hypertension (without complications) (%)	36 (38.7)
Cardiovascular disease (%)	11 (11.8)
Respiratory disease (%)	28 (30.1)
Endocrine (including diabetes) (%)	23 (24.7)
Liver (%)	1 (1.1)
Gastrointestinal (%)	12 (12.9)
Mental health (%)	12 (12.9)
Presence of multimorbidity (2 or more comorbidities) (%)	47 (51.1)
Hospital length of stay, median, days (IQR)	22 (12–55.7)
Critical care length of stay, median, days (IQR)	11.1 (5–25.3)
Acute Physiology and Chronic Health Evaluation II Score, median (IQR)	15 (10–20)
Invasive ventilation (%)	63 (67.7)
Continuous positive airway pressure ventilation (never received invasive ventilation) (%)	16 (17.2)
Continuous positive airway pressure ventilation (also received invasive ventilation) (%)	11 (11.8)
Renal replacement therapy (%)	18 (19.4)
Advanced cardiovascular support (%)	35 (37.6)
Proned (%)	36 (38.7)
Socioeconomic status: (SIMD category)	
1 (most deprived)	30 (32.3)
2	19 (20.4)
3	20 (21.5)
4	8 (8.6)
5 (least deprived)	16 (17.2)
Employment status before admission	
Employed (%)	67 (72)
Unemployed (%)	6 (6.5)
Retired (%)	17 (18.3)
Unknown	3 (3.2)

SIMD, Scottish Index of Multiple Deprivation.

### COVID-19 cohort: patient outcomes

#### Psychosocial outcomes

In the COVID-19 cohort, 67 (72%) patients were employed before admission to critical care, six (6.5%) were unemployed and 17 (18.3%) were retired. Pre and post critical illness employment data were not available for three (3.2%) patients. The distribution of pre-critical illness employment status across the SIMD quintiles is shown in online supplemental file S4. At the time of follow-up, 36 (53.7%) of those working beforehand had returned to employment, one (1.5%) had newly retired and 30 (44.8%) of those who had been employed before critical care had not returned to employment. The six patients who were unemployed before admission, remained unemployed at follow-up.

10.1136/bmjresp-2021-001080.supp4 Supplementary data

**Figure 2:**
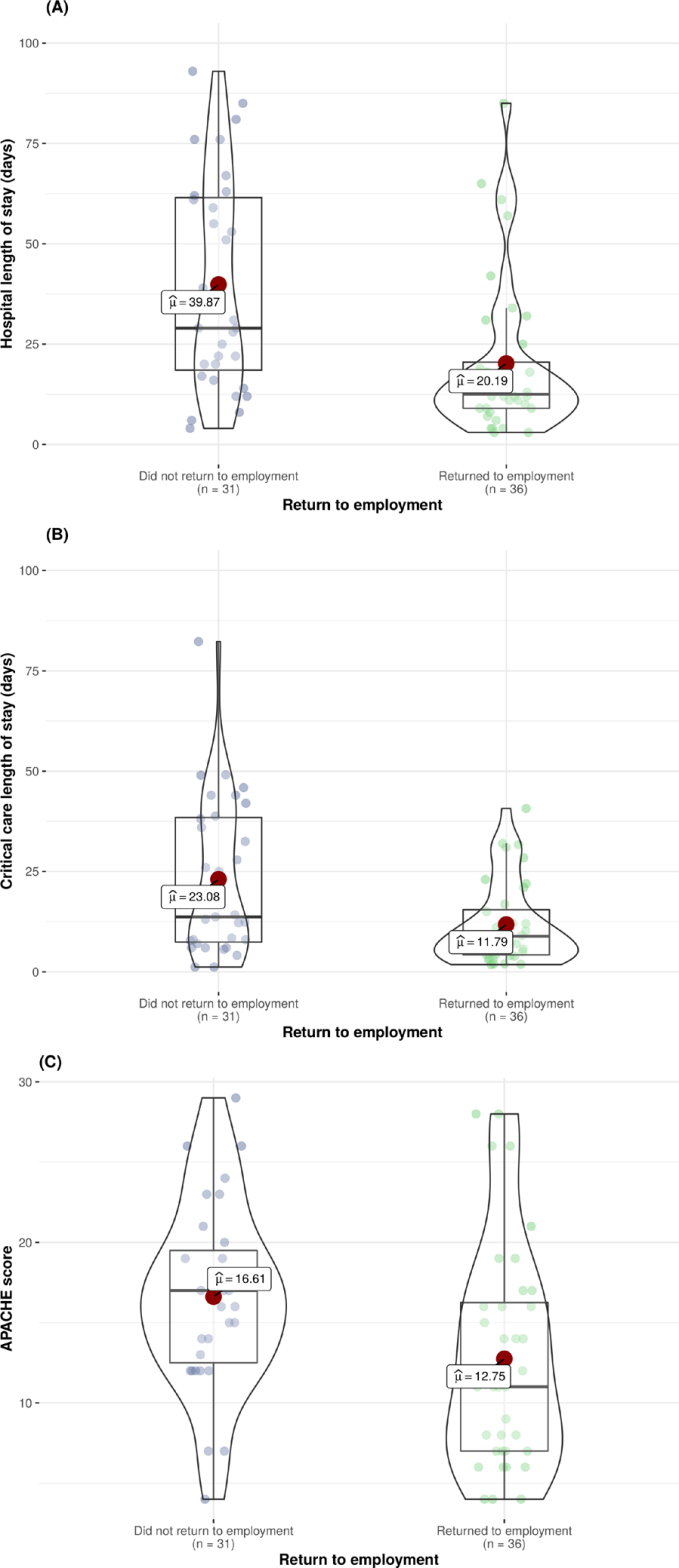
Distribution of hospital (A) and critical care (B) length of stay and APACHE II scores (C) in those returning to, and not returning to employment. APACHE II, Acute Physiology and Chronic Health Evaluation II.

**Figure 3:**
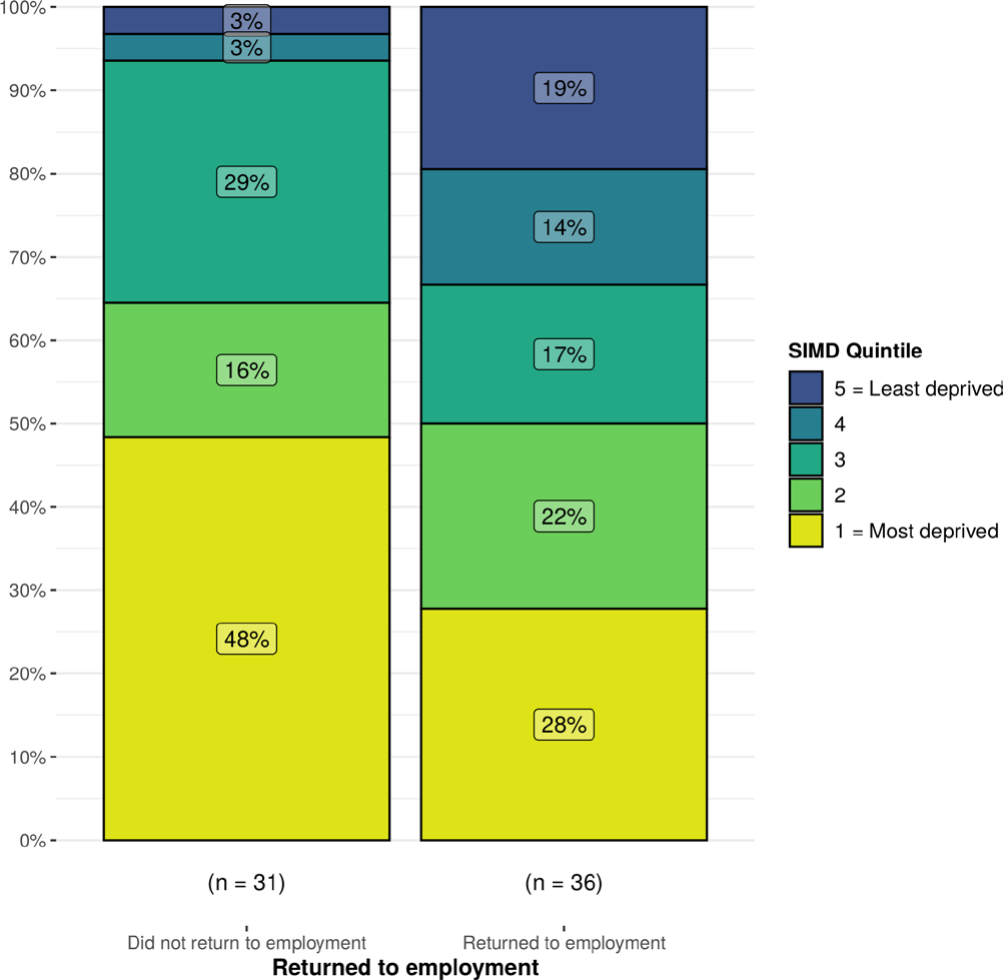
Breakdown in return to baseline employment status across the SIMD quintiles. SIMD, Scottish Index of Multiple Deprivation.

We undertook a subgroup analysis to explore outcomes in those employed before admission to critical care (n=67). Patients who returned to employment had significantly shorter hospital (12.5 days (IQR: 9.0–22.5) vs 29 days (IQR: 17.5–61.8), p<0.01) and critical care (8.9 days (IQR: 4.1–16.1) vs 13.7 days (IQR: 7.1–38.7), p=0.01) stays. Patients who returned to employment also had significantly lower APACHE II scores (11 (IQR:7.0–16.6) vs 17 (12.2–19.8), p=0.01) (figure 2). In this subgroup analysis, there was a significant difference in return to employment across the socio-economic gradient, with a higher proportion of patients from deprived areas failing to return to employment (SIMD 1=48% vs SIMD 5=3%) (figure 3). After adjustment for multimorbidity, follow-up time, age and critical care length of stay, those residing in the most deprived communities were less likely to return to employment in comparison to those from the least deprived communities (OR 0.06; 95% CI 0.00 to 0.76, p=0.03) (online supplemental file S5).

10.1136/bmjresp-2021-001080.supp5 Supplementary data

Twelve (12.9%) patients had pre-existing or historical mental health issues at the time of admission to critical care. Measured via the HADS, 43 (46.2%) patients had symptoms of anxiety. Using previously defined cut-offs (online supplemental file S2), 11 (25.6%) patients had symptoms of mild anxiety, 15 (34.9%) moderate and 17 (39.5%) severe. Symptoms of depression, measured via the HADS, were reported in 32 (34.4%) at follow-up. Eleven (34.4%) patients were classified as having mild depressive symptoms, 16 (50%) moderate and 5 (15.6%) severe. Of those 81 patients, with no history of mental health issues, 35 (43.2%) developed symptoms of anxiety and 24 (29.6%) developed symptoms of depression.

### Health-related quality of life

The median HUS in this cohort was 0.648 (IQR:0.406–0.823) and the median VAS 70 (10-100); 87 (93.5%) patients experienced one or more problem in any EQ-5D-5L domain (table 2). At the time of follow-up, 49 (52.7%) patients described problems with mobility, with 37 (75.5%) of these patients describing these mobility problems as either moderate or extreme. Twenty-seven (29%) patients had problems with self-care activities and 68 (73.1%) had problems carrying out usual activities, such as work and housework. Patients were asked about ongoing breathlessness and fatigue: 73 (78.5%) reported ongoing breathlessness (at rest or on exertion) and 65 (69.9%) described fatigue.

**Table 2:** Breakdown of patient EQ-5D-5L domains and anatomical sites of pain described via the BPI (COVID-19 cohort)

Outcome characteristic	n=93
EQ-5D-5L	
EQ-5D-5L: mobility	
No problem	44 (47.3)
Slight problem	12 (12.9)
Moderate problem	22 (23.7)
Severe problem	15 (16.1)
Extreme problem	0 (0)
EQ-5D-5L: self-care	
No problem	66 (71)
Slight problem	14 (15)
Moderate problem	9 (9.7)
Severe problem	4 (4.3)
Extreme problem	0 (0)
EQ-5D-5L: usual activities	
No problem	25 (26.8)
Slight problem	24 (25.8)
Moderate problem	29 (31.2)
Severe problem	13 (14)
Extreme problem	2 (2.2)
EQ-5D-5L: pain/discomfort	
No problem	27 (29.1)
Slight problem	19 (20.4)
Moderate problem	24 (25.8)
Severe problem	20 (21.5)
Extreme problem	3 (3.2)
EQ-5D-5L: anxiety/depression	
No problem	33 (35.5)
Slight problem	22 (23.7)
Moderate problem	19 (20.4)
Severe problem	15 (16.1)
Extreme problem	4 (4.3)
BPI*	
Pain location from BPI	
Head/neck	8
Anterior trunk	18
Back	20
Arms	26
Legs	38

*Sixty-five patients described pain via the BPI. Patients could describe more than one site of pain.

BPI, Brief Pain Inventory.

We undertook a predefined subgroup analysis to explore the HRQoL (n=67) in those patients who were employed before the critical care admission (n=67). Those who returned to employment had significantly higher HRQoL measured via the VAS (70 (IQR: 54.2–87.9 vs 60 (IQR: 31.7–70), p=0.01), in comparison to those patients who did not return to employment.

### Pain

Sixty-five (69.9%) patients described new pain since discharge from hospital via the BPI, most commonly reported in legs and arms. The distribution of anatomical regions where patients experienced pain is shown in table 2. Pain intensity at its worst in the previous 24 hours, measured via a scale of 0–10 (0=no pain, 10=pain as bad as you can imagine), was more than or equal to 7, in 27 (41.5%) patients (representing severe pain). Pain on average, measured using the same scale, was a median of 5 (IQR: 3–6) across all 65 patients with pain. Almost two-thirds (64.6%) of the patients who reported new pain following severe COVID-19 infection, were taking regular analgesia. Of the patients who had ongoing pain, 61 (94%) patients reported that this pain interfered with aspects of their life such as sleep (80%), mood (66.2%) and relations with other people (47.7%).

### Propensity score matching: COVID-19 cohort versus non-COVID-19 cohort outcomes

Employment, fatigue and breathlessness data was not collected for the non-COVID-19 cohort and thus is not included in the propensity score match analysis.

**Figure 4:**
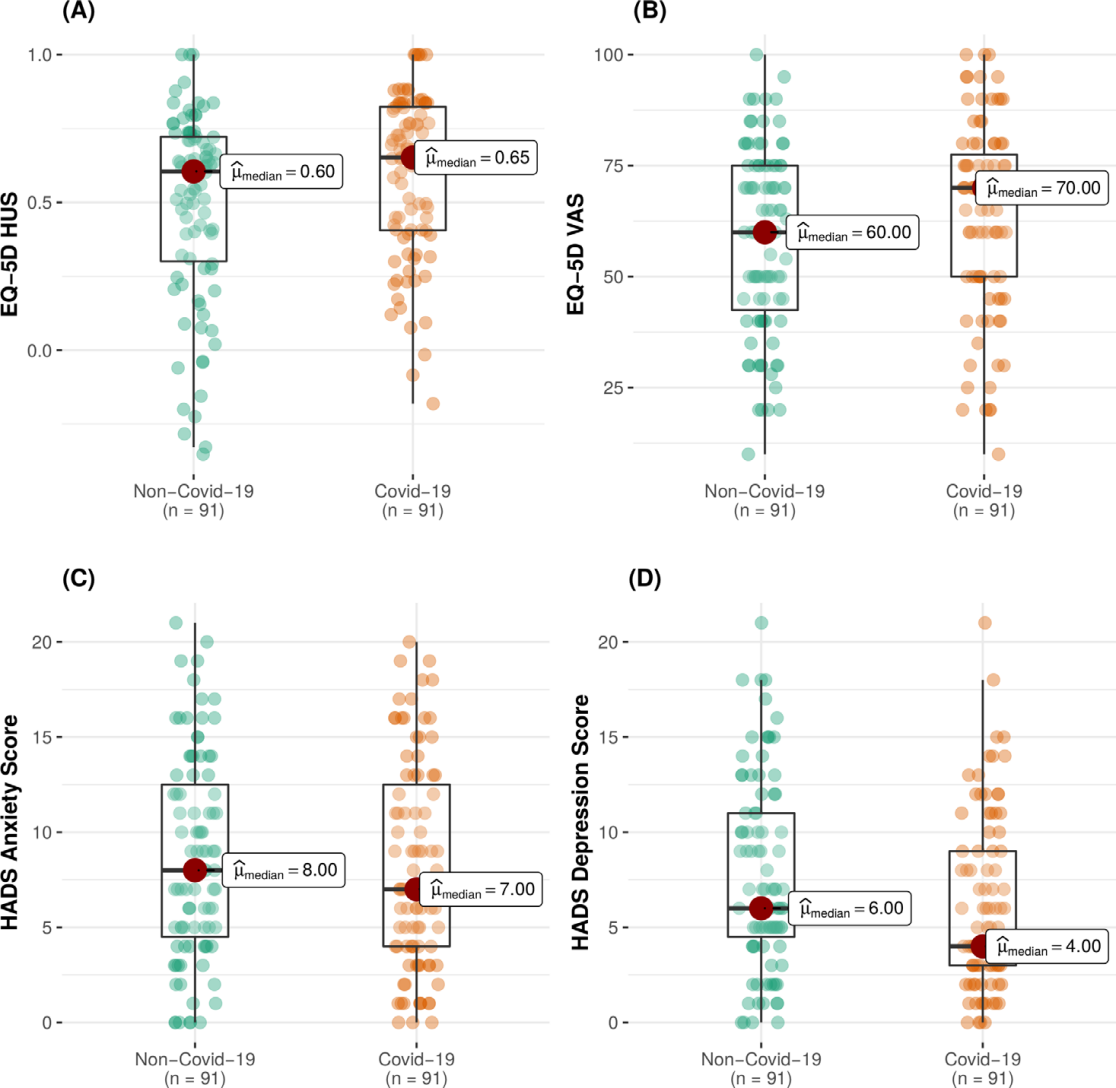
Distribution of EQ-5D (HUS) (A) EQ-5D VAS (B) HADS anxiety (C) and HADS depression (D) across the COVID-19 and the non-COVID-19 cohorts. HADS, Hospital Anxiety and Depression Score; HUS, Health Utility Score; VAS, Visual Analogue Scale.

In total, we matched 91 (97.8%) of the COVID-19 cohort with 91 non-COVID-19 critically ill patients (non-COVID-19 cohort), who attended follow-up. Baseline demographics of the cohorts, pre and post matching, are shown in table 3. There was no significant difference in any outcome measured between the two patient cohorts (COVID-19 vs non-COVID-19 cohorts) in the adjusted analysis (figure 4). Full outputs of the unadjusted and adjusted analysis are shown in online supplemental file S6.

10.1136/bmjresp-2021-001080.supp6 Supplementary data

**Table 3:** COVID-19 and non-COVID-19 cohorts, unmatched and matched datasets

Characteristic/outcome	Pre-matching			Post-matching		
	Non-COVID-19	COVID-19	P value	Non-COVID-19	COVID-19	P value
No of patients	206	93		91	91	
Age, median (IQR), years	58.2 (50–65.7)	59 (54–67)	0.11	59.3 (52.5–66.7)	60 (54–67)	0.74
Gender, male (%)	112 (54.6)	61 (65.6)	0.08	56 (61.5)	61 (67)	0.44
Follow-up time, days, median, (IQR)	144 (96–181)	113 (84–156)	0.05	118 (86–166)	142 (94–180)	0.21
Documented obesity on admission, no (%)	50 (24.4)	30 (32.3)	0.16	24 (26.4)	29 (31.9)	0.41
Prescence of 2 of multimorbidity (two or more comorbidities) (%)	64 (31.2)	47 (51.1)	<0.01	32 (35.2)	45 (50)	0.12
Mental Health problems pre-ICU, no (%)	68 (33.2)	12 (12.9)	<0.01	15 (16.5)	11 (12.1)	0.40
Critical Care Length of stay, median (IQR) days	11.6 (5–25.3)	11.1 (5–25.3)	0.67	12.8 (7–23.7)	11.1 (5–25.8)	0.21
APACHE II, median (IQR)	20 (15–25)	15 (10–20)	<0.01	17 (13–20.1)	16 (12–19)	0.08
Socioeconomic status (SIMD category)			0.13			0.71
1 (most deprived)	83 (40.5)	29 (31.5)		33 (36.2)	28 (31.1)	
2	50 (24.4)	19 (20.7)		16 (17.6)	19 (21.1)	
3	31 (15.1)	20 (21.7)		21 (23.1)	20 (22.2)	
4	22 (10.7)	8 (8.7)		10 (11)	7 (7.8)	
5	19 (9.3)	16 (17.4)		11 (12.1)	16 (17.8)	

APACHE II, Acute Physiology and Chronic Health Evaluation II; SIMD, Scottish Index of Multiple Deprivation.

## Discussion

This multicentre study has revealed that survivors of severe COVID-19 infection experience longer-term physical, emotional and social problems. To our knowledge, this is the first study which has described the outcomes of severe COVID-19 survivors in relation to non-COVID-19, critical care survivors. In this study, those patients who have been critically unwell due to COVID-19 appear to have similar outcomes to other ICU survivor cohorts.^24 25^ The high levels of new unemployment seen in the post hospital discharge period are also common in ICU survivors.^26^ In the context of the COVID-19 pandemic, new unemployment is likely to be multifactorial and not solely related to new onset frailty or ill health. However, its distribution across the socioeconomic gradient is worrying.

The disproportionate impact of COVID-19 on the most deprived communities has been described previously. Those from socioeconomically deprived areas are more likely to develop COVID-19 and die from the disease.^23 27^ This study suggests that there may also be worse long-term outcomes for people from the most deprived communities. Although return to employment may not be viewed as a traditional health metric, societal factors outside healthcare undoubtedly contribute to health.^28^ Previous global events such as the 2008 financial crisis, led to a deterioration in population-level mental health.^29^ It is essential policy makers, alongside healthcare providers, ensure all is done to maximise outcomes for those most at risk, by ensuring sufficient social and economic security.^30–32^

Over two-thirds of patients in this cohort reported new onset pain following severe COVID-19 infection, a prevalence consistent with previous critical care research and the non-COVID-19 cohort examined in this study.^33^ Patients described this pain, in many cases as severe, regularly interfering with different aspects of activities of daily living. More work which examines the mechanisms driving this reported pain is required.

The results of this study demonstrate that structured multidisciplinary rehabilitation after severe illness, cannot be viewed as a luxury. Standardised rehabilitation pathways which aim to improve global HRQoL should be universally implemented. For those who cannot return to work, structured, cohesive vocational rehabilitation should be provided to ensure that those recovering have purposeful and meaningful activity. Those currently designing treatment pathways for COVID-19 must ensure that rehabilitation is not only multidisciplinary, but also multiagency, crossing health and social care boundaries.^34^

## Limitations

This study has limitations inherent in most follow-up studies in this field. Epidemiological studies have demonstrated that there is a high level of anxiety at a population level due to the ongoing pandemic; these factors could have influenced the emotional outcomes reported in the COVID-19 cohort.^33^ Moreover, we have limited data on patient mental health status before the pandemic. As such, some of the problems detailed in this analysis may not be related to the critical care experience. The decreased employment figures shown are also likely to be multifactorial, due to the high levels of unemployment internationally. We also have no information on plans to return to employment, or data on job seeking intention. Additionally, we only have data at one time point; the recovery trajectory for each individual participant is likely to be variable. Longitudinal work which examines the dynamics of these outcomes is needed. Moreover, employment, breathlessness and fatigue data were not available for the non-COVID-19 cohort.

We recognise that our use of a historical control has limitations. Moreover, although both patient cohorts attended the same recovery programme, the societal changes and the social support available during the COVID-19 pandemic, were distinctly different and may have influenced the results reported.

We specifically sought to understand new pain since the COVID-19 illness; however, patients may have reported pre-existing problems or the exacerbation of previous aliments. Although we have basic baseline data about those who did not attend the clinic, this works lacks detailed information about this group. Those who did not attend InS:PIRE, could have had a different recovery experience. Finally, due to the small sample size, caution must be taken when interpreting these results. This analysis requires replication with a larger patient population.

## Conclusions

In conclusion, patients who have been severely ill due to COVID-19 experience similar problems to other ICU survivors after discharge. In this multicentre study, lower numbers of patients from socioeconomically deprived areas returned to work following severe COVID-19, compared with their more affluent counterparts. This study highlights the need for multifaceted rehabilitation, which focuses on all aspects of health and well-being.

## Data Availability

Data are available on reasonable request. The datasets used and/or analysed during the current study are available from the corresponding author on reasonable request.
