# Supplementary material for: Long-term outcomes following severe COVID-19 infection: a propensity matched cohort study
Source: BMJ Open Respir Res. 2021 Dec 9;8(1):e001080. doi: 10.1136/bmjresp-2021-001080 (PMC8663070; doi:10.1136/bmjresp-2021-001080)
Supplement: Supplementary data [file bmjresp-2021-001080supp001.pdf]

S1: Information on missing data, COVID-19 and non-COVID-19 cohorts

	COVID-19 COHORT	NON COVID-19 COHORT
**Characteristic**	n=93	n=206
**Gender, male (%)**	0 (0)	1 (0.5)
**Age, median years (IQR)**	0 (0)	1 (0.5)
**Obesity (%)**	0 (0)	1 (0.5)
**Black and Minority Ethnic (%)**	0 (0)	N/A
**Smoking (%)**	0 (0)	N/A
**Comorbidities:**		
Hypertension (without complications) (%)	0 (0)	1 (0.5)
Cardiovascular disease (%)	0 (0)	1 (0.5)
Respiratory disease (%)	1 (1.1)	1 (0.5)
Endocrine (including diabetes) (%)	0 (0)	1 (0.5)
Liver (%)	0 (0)	1 (0.5)
Gastrointestinal (%)	0 (0)	1 (0.5)
Mental Health (%)	0 (0)	1 (0.5)
Presence of Multimorbidity (2 or more comorbidities) (%)	0 (0)	0 (0)
**Hospital length of stay, median, days (IQR)**	12 (13)	1 (0.5)
**Critical care length of stay, median, days (IQR)**	0 (0)	1 (0.5)
**Acute Physiology and Chronic Health Evaluation II Score, median (IQR)**	18 (19.3)	3 (1.5)
**Invasive ventilation (%)**	1 (1.1)	1 (0.5)
**Continuous Positive Airway Pressure ventilation (never received invasive ventilation) (%)**	0 (0)	N/A
**Continuous Positive Airway pressure ventilation (also received invasive ventilation) (%)**	1 (1.1)	N/A
**Renal Replacement Therapy (%)**	0 (0)	1 (0.5)
**Advanced Cardiovascular Support (%)**	0 (0)	1 (0.5)
**Proned (%)**	1 (1.1)	N/A
**Socio-economic status: (SIMD category)**	1 (1.1)	1 (0.5)
**Employment status before admission:**	3 (3.2)	N/A
**EQ-5D-5L (Health Utility)**	2 (2.2)	15 (7.3)
**EQ-ED-EL (VAS)**	2 (2.2)	24 (11.7)
**HADS (Depression)**	1 (1.1)	1 (0.5)
**HADS (Anxiety)**	0 (0)	1 (0.5)
