# Supplementary material for: Long-term outcomes following severe COVID-19 infection: a propensity matched cohort study
Source: BMJ Open Respir Res. 2021 Dec 9;8(1):e001080. doi: 10.1136/bmjresp-2021-001080 (PMC8663070; doi:10.1136/bmjresp-2021-001080)
Supplement: Supplementary data [file bmjresp-2021-001080supp002.pdf]

S2

Interpretation of HADS score (Zigmond and Snaith 1983)

Score	Interpretation
**0-7**	Normal
**8-10**	Mild
**11-14**	Moderate
**15-21**	Severe
