# Supplementary material for: Long-term outcomes following severe COVID-19 infection: a propensity matched cohort study
Source: BMJ Open Respir Res. 2021 Dec 9;8(1):e001080. doi: 10.1136/bmjresp-2021-001080 (PMC8663070; doi:10.1136/bmjresp-2021-001080)
Supplement: Supplementary data [file bmjresp-2021-001080supp003.pdf]

S3

Characteristics of study participants and those patients who were invited, but did not attend InS:PIRE

**Characteristic**	**InS:PIRE cohort (n=93)**	**Invited, but did not attend (n=76)**
Gender, male (%)	61 (65.6)	51 (67.1)
Age, year, median (IQR)	59 (54-67)	53 (45-60)
Critical Care Length of Stay, days, median (IQR)	11.1 (5-25.3)	3.9 (2.3-9.2)
Socio-economic status: SIMD category:		
1 (most deprived)	30 (32.3)	31 (40.8)
2	19 (20.4)	15 (19.7)
3	20 (20.5)	12 (15.8)
4	8 (8.6)	8 (10.5)
5 (least deprived)	16 (17.2)	10 (13.2)
APACHE II, median (IQR)	15 (10-20)	13.5 (9-16)
