# Supplementary figures and images for: Long-term outcomes following severe COVID-19 infection: a propensity matched cohort study
Source: BMJ Open Respir Res. 2021 Dec 9;8(1):e001080. doi: 10.1136/bmjresp-2021-001080 (PMC8663070; doi:10.1136/bmjresp-2021-001080)

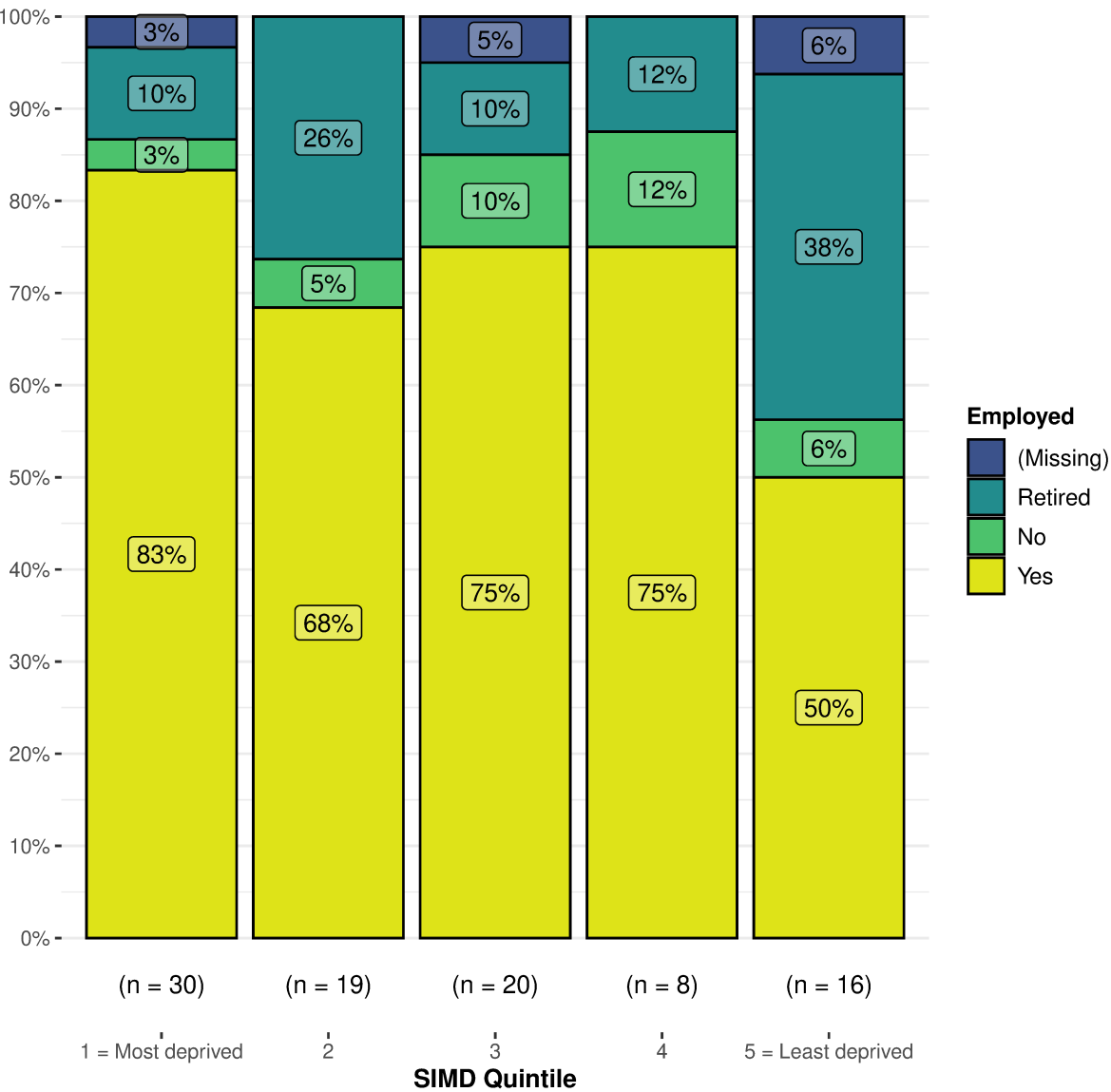

Supplement: Supplementary data [file bmjresp-2021-001080supp004.pdf]
