# Supplementary material for: Long-term outcomes following severe COVID-19 infection: a propensity matched cohort study
Source: BMJ Open Respir Res. 2021 Dec 9;8(1):e001080. doi: 10.1136/bmjresp-2021-001080 (PMC8663070; doi:10.1136/bmjresp-2021-001080)
Supplement: Supplementary data [file bmjresp-2021-001080supp005.pdf]

S5

Logistic Regression Model: Failure to Return to Employment (n=67)

**Variable**	**Adjusted Odds Ratio (95% CI)**	**p value**
**SIMD 1 (Reference)**	1	
SIMD 2	0.66 (0.14-3.14)	0.61
SIMD 3	2.05 (0.41-10.30)	0.39
SIMD 4	0.22 (0.02-2.63)	0.24
SIMD 5 (Least Deprived)	0.06 (0.00-0.76)	**0.03**
Multimorbidity (presence of 2 or more comorbidities)	1.13 (0.27-4.82)	0.87
Critical care length of stay	1.05 (0.99-1.10)	0.07
Age	1.06 (0.99-1.12)	0.08
Follow-up time (days)	0.99 (0.97-1.00)	0.08

McPeake J, *et al. BMJ Open Resp Res* 2021; 8:e001080. doi: 10.1136/bmjresp-2021-001080
