# Supplementary material for: Long-term outcomes following severe COVID-19 infection: a propensity matched cohort study
Source: BMJ Open Respir Res. 2021 Dec 9;8(1):e001080. doi: 10.1136/bmjresp-2021-001080 (PMC8663070; doi:10.1136/bmjresp-2021-001080)
Supplement: Supplementary data [file bmjresp-2021-001080supp006.pdf]

S6: Matched, Unadjusted and Adjusted Modelling outputs (COVID-19 vs non-COVID-19 cohorts)

EQ-5D-5L Health Utility Score (Linear Regression)

Variable	Unadjusted Estimate (95% CI)	P value	Adjusted Estimate (95% CI)	P value
Cohort difference	0.09 (-0.005-0.18)	0.06	0.08 (-0.005-0.17)	**0.06**
Age (Years)	0.002 (-0.002-0.006)	0.24	0.001 (-0.004-0.006)	0.66
Gender (Male)	0.03 (-0.06-0.13)	0.51	0.006 (-0.09-0.10)	0.90
ICU Length of Stay	-0.002(-0.005-0.001)	0.20	-0.001 (-0.004-0.002)	0.34
APACHE II	0.005 (-0.004-0.013)	0.26	0.006 (-0.004-0.016)	0.24
Obesity	-0.09 (-0.191-0.007)	0.70	-0.037 (-0.14-0.067)	0.48
Follow-up time	-0.0004 (-0.001-0.000)	**0.03**	-0.0002(-0.006-0.001)	0.17
SIMD 2	-0.04 (-0.17-0.09)	0.57	-0.03 (-0.158-0.09)	0.58
SIMD 3	0.062 (-0.06-0.18)	0.32	0.08 (-0.03-0.21)	0.14
SIMD 4	0.12 (-0.04-0.29)	0.13	0.12 (-0.03-0.28)	0.12
SIMD 5	0.13 (-0.006-0.27)	0.06	0.096 (-0.036-0.229)	0.15
Presence of one comorbidity	-0.007 (-0.13-0.12)	0.92	-0.029 (-0.160-0.101)	0.66
Presence of two or more comorbidities	-0.04 (-0.15-0.06)	0.44	-0.09 (-0.199-0.021)	0.11
Previous Mental Health problems (pre critical care)	**-0.22 (-0.34- -0.01)**	**<0.001**	**-0.191 (-0.319- -0.162)**	**0.004**

EQ-5D-5L Visual Analogue Scale (Linear Regression)

Variable	Unadjusted Estimate (95% CI)	P value	Adjusted Estimate (95% CI)	P value
Cohort difference	4.67 (-1.83-11.17)	0.16	4.73 (-1.12-11.28)	**0.16**
Age (Years)	0.08 (-0.19-0.36)	0.55	-0.02 (-0.33-0.31)	0.92
Gender (Male)	2.86 (-3.82-9.55)	0.40	0.76 (-6.13-7.66)	0.83
ICU Length of Stay	-0.03 (-0.24-0.19)	0.81	0.02 (-0.18-0.23)	0.84
APACHE II	0.32 (-0.27-0.91)	0.29	0.41 (-0.29-1.10)	0.25
Obesity	-6.73 (-13.6-0.13)	0.05	-4.03 (-11.16-3.1)	0.27
Follow-up time	-0.007 (-0.03-0.02)	0.61	0.002 (-0.02-0.03)	0.86
SIMD 2	-1.4(-10.73-7.93)	0.77	-0.84 (-10.05-8.36)	0.86
SIMD 3	2.49 (-6.34-11.33)	0.58	4.55 (-4.25-13.36)	0.31
SIMD 4	1.34 (-10.39-13.07)	0.82	0.87 (-10.72-12.46)	0.88
SIMD 5	9.75 (-0.07-19.57)	0.05	7.96 (-1.72-17.63)	0.11
Presence of one comorbidity	-1.63 (-10.53-7.28)	0.72	-3.43 (-12.75-5.89)	0.47
Presence of two or more comorbidities	-2.77 (-10.46-4.93)	0.48	-5.40 (-13.56-2.76)	0.19
Previous Mental Health problems (pre critical care)	**-15.57 (-24.7- -6.44)**	**0.001**	**-14.71 (-24.32- -5.10)**	**0.003**

Hospital Anxiety and Depression Scale- Anxiety Component (Linear Regression)

**Variable**	**Unadjusted Estimate (95% CI)**	**P value**	**Adjusted Estimate (95% CI)**	**P value**
Cohort difference	-0.30 (-1.99-1.38)	0.72	-0.07 (-1.162-1.48)	**0.93**
Age (Years)	**-0.13 (-0.19- -0.06)**	**<0.001**	**-0.08 (-0.15- -0.002)**	**0.05**
Gender (Male)	**-1.78 (-3.48- -0.09)**	**0.04**	**-1.02 (-2.65-0.61)**	**0.21**
ICU Length of Stay	-0.03 (-0.08-0.03)	0.35	-0.03 (-0.08-0.02)	0.26
APACHE II	**-0.22 (-0.37- -0.07)**	**0.005**	-0.15 (-0.31-0.01)	0.07
Obesity	0.92 (-0.85-2.68)	0.31	0.24 (-1.50-1.98)	0.79
Follow-up time	0.004 (-0.002-0.01)	0.19	0.003 (-0.003-0.009)	0.35
SIMD 2	0.45 (-1.90-2.80)	0.70	0.14 (-1.98-2.26)	0.90
SIMD 3	-1.37 (-3.61-0.86)	0.23	-2.05 (-4.08- -0.011)	0.05
SIMD 4	-1.30 (-4.26-1.67)	0.39	-1.04 (-3.76-1.68)	0.45
SIMD 5	**-2.89 (-5.41- -0.37)**	**0.03**	-2.05 (-4.34-0.24)	0.08
Presence of one comorbidity	-1.41 (-3.57-0.74)	0.20	0.29 (-1.88-2.45)	0.79
Presence of two or more comorbidities	-1.40 (-3.33-0.53)	0.15	0.36 (-1.56-2.28)	0.71
Previous Mental Health problems (pre critical care)	**4.68 (2.49-6.87)**	**<0.001**	**4.14 (1.94-6.34)**	**0.0003**

Hospital Anxiety and Depression Scale- Depression Component (Linear Regression)

**Variable**	**Unadjusted Estimate (95% CI)**	**P value**	**Adjusted Estimate (95% CI)**	**P value**
Cohort difference	-1.28 (-2.76-0.20)	0.09	-1.09 (-2.43-0.25)	**0.11**
Age (Years)	**-0.07 (-0.13- -0.01)**	**0.03**	-0.03 (-0.01-0.03)	0.31
Gender (Male)	-1.07 (-2.56-0.42)	0.16	-0.34 (-1.79-1.10)	0.64
ICU Length of Stay	-0.01 (-0.06-0.04)	0.59	-0.03 (-0.07-0.02)	0.25
APACHE II	**-0.16 (-0.29- -0.03)**	**0.02**	-0.15 (-0.30-0.004)	0.06
Obesity	1.30 (-0.32-2.92)	0.12	0.43 (-1.12-1.98)	0.58
Follow-up time	0.006 (0-0.011)	0.05	0.003 (-0.002-0.008)	0.22
SIMD 2	0.04 (-2.06-2.14)	0.97	-0.19 (-2.03-1.65)	0.83
SIMD 3	-0.57 (-2.54-1.40)	0.57	-1.21 (-2.97-0.54)	0.17
SIMD 4	-0.64 (-3.29-2.0)	0.63	-0.56 (-2.99-1.87)	0.65
SIMD 5	**-2.58 (-4.82- -0.35)**	**0.02**	-1.97 (-3.97-0.04)	0.05
Presence of one comorbidity	0.55 (-1.43-2.53)	0.59	1.62 (-0.26-3.51)	0.09
Presence of two or more comorbidities	0.26 (-1.48-1.99)	0.77	1.51 (-0.16-3.19)	0.08
Previous Mental Health problems (pre critical care)	**5.22 (3.27-7.17)**	**<0.001**	**4.97 (2.90-7.05)**	**<0.001**

EQ-5D-5L: Pain analysis (Logistic Regression) *

**Variable**	**Unadjusted Estimate (95% CI)**	**P value**	**Adjusted Estimate (95% CI)**	**P value**
Cohort difference	0.04 (-0.66-0.75)	0.90	0.038 (-0.74-0.82)	**0.92**
Age (Years)	-0.02 (-0.04-0.01)	0.31	-0.02 (-0.06-0.02)	0.33
Gender (Male)	-0.06 (-0.74-0.73)	0.99	0.01 (-0.82-0.85)	0.98
ICU Length of Stay	0.02 (-0.002-0.04)	0.08	0.02 (-0.01-0.004)	0.14
APACHE II	-0.02 (-0.08-0.05)	0.65	-0.01 (-0.11-0.08)	0.77
Obesity	0.54 (-0.21-1.28)	0.16	0.28 (-0.60-1.15)	0.53
Follow-up time	0.002 (-0.001-0.004)	0.20	0.01 (-0.001-0.004)	0.37
SIMD 2	0.20 (-0.75-1.15)	0.68	0.23 (-0.76-1.23)	0.64
SIMD 3	-0.74 (-1.78-0.30)	0.16	-0.95 (-2.09-0.20)	0.10
SIMD 4	-0.69 (-2.08-0.69)	0.33	-0.62 (-2.08-0.84)	0.40
SIMD 5	-0.64 (-1.78-0.5)	0.27	-0.49 (-1.7-0.73)	0.43
Presence of one comorbidity	0.02 (-1.02-1.06)	0.97	0.12 (-1.08-1.32)	0.84
Presence of two or more comorbidities	0.38 (-0.45-1.20)	0.37	0.69 (-0.30-1.68)	0.17
Previous Mental Health problems (pre critical care)	0.66 (-0.23-1.55)	0.15	0.54 (-0.48-1.57)	0.30

*dichotomised at severe pain and above in the EQ-5D-5L scale
